# Colloidal Nanosilica Treatments for Sealing Cracks in Mortar

**DOI:** 10.3390/ma15186338

**Published:** 2022-09-13

**Authors:** M. Sánchez-Moreno, J.L. García-Calvo, F. Tavares-Pinto

**Affiliations:** 1 Departamento de Química Inorgánica e Ingeniería Química, Instituto de Investigación en Nanoquímica, IUNAN, Universidad de Córdoba, 14014 Córdoba, Spain; 2 Institute for Construction Sciences “Eduardo Torroja” (IETcc-CSIC), C/Serrano Galvache 4, 28033 Madrid, Spain; 3 Departamento de Ingeniería Mecánica, Universidad de Córdoba, 14014 Córdoba, Spain

**Keywords:** colloidal nanosilica, cracked mortar, sealing cracks, healing ability, C/S ratio, C-S-H gels

## Abstract

Presence of microcracks in concrete can diminish the service life of a structure. The injection of materials for filling the crack is proposed for facing this problem. The traditional materials used for sealing cracks present some drawbacks, such as the difficulties of inorganic materials for flowing to all the depth of the crack and the lack of compatibility with the cementitious matrix in the case of organic materials. In this work, the injection of colloidal nanosilica dispersed in water is proposed for filling microcracks in mortars. The effect of the injection procedure on the sealing performance of the colloidal nanosilica has been assessed. The ability of colloidal nanosilica for penetrating through the crack and its posterior gelification-solidification inside the crack after a curing period have been confirmed. The microscopic analysis of a cross-section of the crack indicates that the sealing ability of the nanosilica seems to be not only due to the filling of the crack but also to chemical interactions with the cementitious phases of the surrounding crack sides.

## 1. Introduction

Both the structural performance and the durability of reinforced concrete can be affected due to the presence of cracks compromising the service life of these structures. Cracks act as preferential path for the entrance of water and aggressive agents, which could reach the level of the rebar thus promoting its corrosion in shorter times than expected. Then, crack repair becomes an issue of special interest in construction to guarantee the designed service life of concrete structures. To minimize the damage on the structures, an optimal solution is to control and repair cracks at the earliest stages and thus preventive technologies, such as those based in “self-healing” concept, appear as highly interesting innovative alternatives [1]. Several reviews classifying the different approaches [2], evaluating their efficiency [3], and proposing sustainable biobased solutions [4] have been reported.

However, for the case of already existing cracks, when these preventive actions are not appropriate solutions, different repair concepts such as the injection of polymeric materials [5] or the use of epoxy polymers of filling cracks [6] or joints [7] in concrete are conventionally utilized. Moreover, cementitious materials such as high-performance cements [8] and expansive grouts [9,10] are proposed for repairing cracked concrete. These methods allow the physical sealing of the crack, but no healing action is expected. The crack closure by electrodeposition techniques due to the precipitation of chemical compounds filling the crack has been reported [11] and even a numerical has been recently proposed [12]. Nowadays, adapting self-healing concepts for the sealing of existing cracks stands out as promising technologies [13]. Biobased treatments for filling cracks in concrete [14] allow not only a physical sealing but also a healing process on the repaired crack [15]. Recently, the application of crack sealants with added function such as re-alkalinization and re-passivation properties has been proposed [16].

The addition of colloidal nano-silica particles to improve the performance of cementitious systems has been extensively used during the last decade [17] because of its pozzolanic reactivity [18] besides the pore-filling effect [19]. The surface application of colloidal silica nanoparticles has been proposed for improving the resistance of hardened concrete against chloride penetration through the concrete pores [20] and against concrete carbonation [21]. Ethyl-silicate, precursor of nano-SiO_2_, has been reported to have pozzolanic effect when applied as surface treatment on hardened concrete due to the precipitation of silica gel inside the pores [22]. Moreover, nanosilica particles can penetrate through concrete pores promoting the formation of new gels that fill the pores and refine the porous structure of the cementitious matrix [23].

Based on the ability of colloidal nanoparticles for producing a gel able to fill capillary pores in existing cementitious matrixes, in the present work, the application of colloidal nanosilica particles for sealing cracks in hardened mortar samples is evaluated. Different application techniques are assessed depending on the main transport mechanism of the colloidal nanosilica particles: electromigration, injection, and capillary suction. These application techniques aim to simulate the mechanism occurring in the different treatments used for applying external methods for repairing cracked concrete: injection under pressure [5], penetration by gravity [6], and electrodeposition [12].

The sealing and healing abilities of colloidal nanosilica are analyzed by back scattering scanning electronic microscopy (BSEM) and energy dispersive X-ray (EDX) for the elemental analysis of the cementitious matrix.

## 2. Materials and Methods

### 2.1. Materials

The study was carried out on prismatic (40 mm × 40 mm × 160 mm) mortar samples with a cement: sand ratio 1:3 and a water-to-cement ratio of 0.5. Polypropylene microfibers (31 μm diameter, 12 mm long) were also added to the mortar mix (8 kg/m^3^ mortar). CEM I 42.5 R/SR according to EN 197-1 [24] and normalized siliceous sand (0/2 mm) were used for producing the mortar samples. The chemical composition of the cement is detailed in Table 1.

The mortar mixes were prepared according to the Spanish standard EN 196-1: 2018 [25], using 450 g of cement per mold (each mold contained 3 prismatic specimens).

After casting, mortar samples were submitted to moist curing (98% RH, 20 ± 2 °C) for 28 days. Three-point bending flexural tests were used applying a load of 50 ± 10 N/s until the generation of one main crack in the sample. The crack mouth opening displacement (CMOD) was not controlled but similar final strength was applied in all the cases (≈11 MPa); for this reason, different final crack widths were obtained in each case. The crack size in each sample, resumed in Table 2, was measured with the computer software KAPPA Image on photographs obtained with an OLYMPUS DP25 microscope camera. The sealing method used for the treatment of each sample is also indicated in Table 2.

A commercial colloidal nanosilica aqueous dispersion (Bindzil 30/301, 29% wt, 7 nm average particle size) was used for the sealing treatments. Silica particles are amorphous, discrete, spherical, and monodispersed in alkaline aqueous solution. The suspension was applied as received for the crack sealing treatments.

### 2.2. Crack Sealing Treatments

Three different methods for crack repair were evaluated depending on the controlling transport mechanism for the colloidal nanosilica. For the three methods, several applications were considered for establishing wet-dry cycles and thus improving the treatment penetration. The cycles were defined with the criteria of wetting the samples the same number of times. The specific experimental conditions for the three treatments are defined below. The different samples tested by each method are resumed in Table 2.

#### 2.2.1. Electromigration

**Figure 1:**
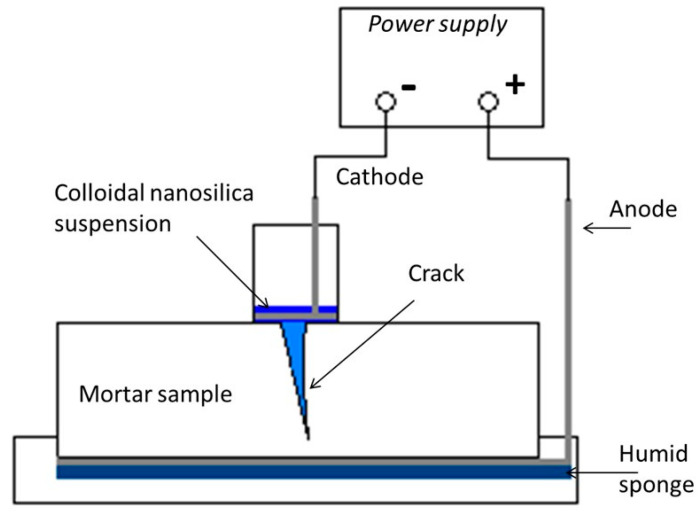
Set-up scheme for crack sealing by the electromigration of colloidal nanosilica particles.

The nanosilica transport takes place under the action of an electric field connected between to electrodes located at both sides of the crack, as schematized in Figure 1.

On the top of the sample, vertically to the crack, a ponding containing the nanosilica dispersion was located and connected to the negative pole (cathode) of the power supplier. The slight negative superficial charge of the nanoparticles will be attracted to the positive electrode. On the bottom, a continuously humid sponge was located in contact with an external electrode connected to the positive pole (anode) of the power supplier. The treatment consisted of three cycles of 2 days of electric field connection (12 VDC) followed by a 5 day disconnection period. Before the treatment, the cracked mortar sample was saturated to guarantee that migration was the main transport mechanism for silica nanoparticles. The saturated conditions were maintained during the whole test.

#### 2.2.2. Injection

The nanosilica dispersion was manually injected into the crack with a syringe. In this case, mortar samples were previously dried during 7 days at laboratory atmosphere (≈20 °C and ≈55%RH) to improve the penetration of the nanosilica suspension during the repair treatment. The total treatment consisted of the application of one injection per day for one week.

#### 2.2.3. Capillary Suction

As in the previous case, before the capillary suction treatment, the mortar samples were dried for 7 days at laboratory conditions to promote the capillary forces to be the main mechanisms for nanosilica particles transport. The whole treatment of capillary suction consisted of three cycles of wetting/drying: two days wetting by locating a ponding containing the nanosilica dispersion vertically to the crack and 5 days drying at the laboratory atmosphere after removing the suspension from the ponding.

### 2.3. Crack Sealing Characterization

In all the studied cases, once the treatment finished, the repaired mortar samples were maintained in humid conditions (98 ± 2% HR, 20–22 °C) for 15 days before the characterization tests. This period was considered for promoting the formation of the silica gel inside the crack and to favor the pozzolanic reactions to take place because of the nanosilica particles transport. One sample of each treatment was subjected to a three-point bending test to evaluate the residual mechanical behaviour after the 15 days conditioning under humid conditions. The sealing characterization was carried out both by indirect and direct measures.

The electrical resistance through the crack was used as indirect measure by comparing the initial and final values, before and after the sealing treatments. Both resistances were measured in saturated conditions to be comparable. A non-destructive measure of the electrical resistance by applying an AC current (0.1 VAC peak to peak at 1 kHz) through the transversal section of the crack was employed.

The superficial aspect of the cracks before and after the treatments was evaluated and the crack width reduction was measured with an optical microscope. The analysis of the microstructure and composition of the mortars were assessed by back scattering scanning electron microscopy (BSEM) coupled to energy dispersive X-ray analysis (EDX). BSEM images were obtained using a scanning electron microscope Hitachi S-4800 equipped with an energy dispersive analyzer BRUKER 5030 that allowed to analyze the chemical composition of the hydration products. For the microscopic characterization, a cross-section of the repaired crack was embedded in epoxy resin, cut, polished, and then coated with carbon.

## 3. Results and Discussion

### 3.1. Electrical Resistance Measurements

**Figure 2:**
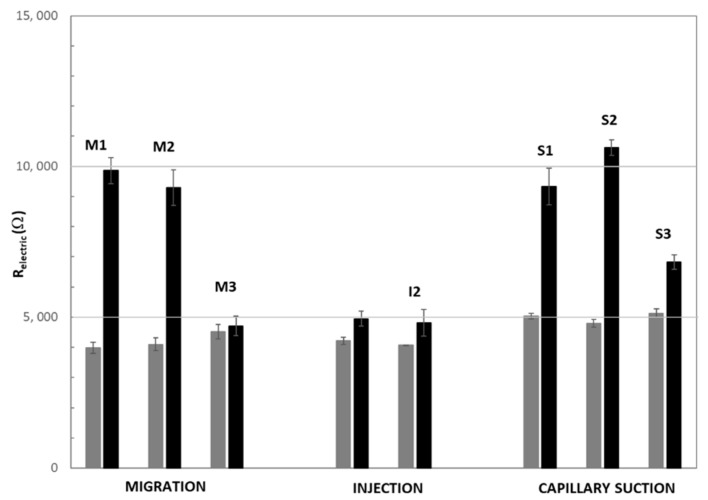
Electrical resistance values before and after applying the sealing treatments.

In Figure 2, the variation of the electrical resistance before and after the treatment has been represented.

Before the treatment, no significant differences were measured in the electric resistance values for the different mortar samples, as can be observed in Figure 2. After the sealing treatment, an increase of the electrical resistance was registered in all the cases. This increase must be related to the sealing of the crack due to the treatment with the colloidal nanosilica and it can be expected that denser sealing will promote a higher increase of the resistance value. From Figure 2, a certain influence of the treatment method can be deduced. In fact, migration and capillary suction treatments seem to be more effective than the direct nanosilica injection in the crack.

However, the crack width has also a significant influence on the treatment effectiveness. In fact, the higher electric resistance values after the sealing treatment are registered in the case of the narrower cracks, corresponding to M1, S1, and S2 (see Table 1). The change in the electric resistance values for the larger cracks, M3 or I2, is not very significant. When samples with similar crack size but exposed to different type of treatment are compared, no significant influence of the type of treatment can be observed. Samples with narrow cracks such as M1, exposed to migration treatment, and S1, exposed to capillary suction treatment, show similar electrical resistance values after the treatment, suffering a significant increase. However, in samples with large cracks, such as M3 and I1, no significant increase of the electrical resistance values is obtained after the treatment.

### 3.2. Visual Inspection of Treated Cracks

**Figure 3:**
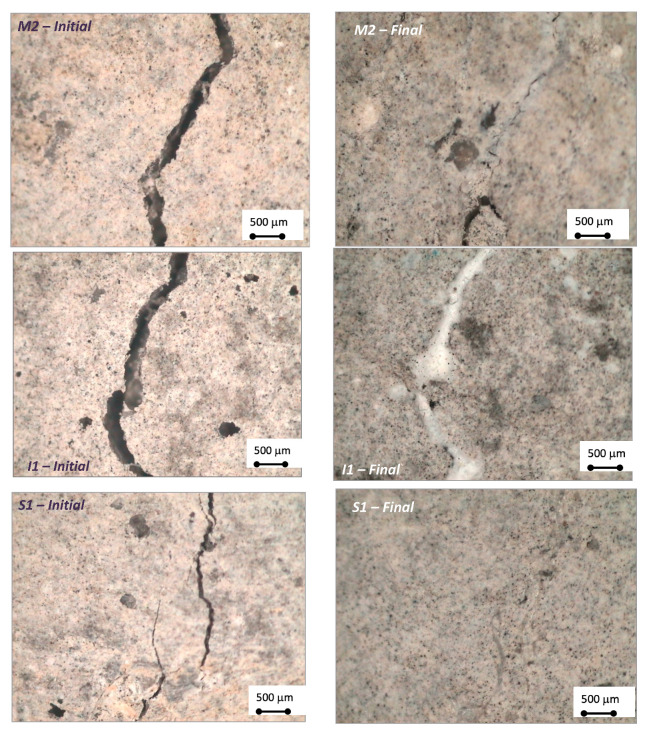
Magnified view of the cross-section of the crack surface before and after the different sealing treatments.

The visual observation of the samples after the treatment with the colloidal silica nanoparticles was carried out with an optical microscope. In Figure 3, the comparison between the initial and the final state of the cross section of the crack after each treatment has been included (one example for each method). In all cases, cracks were filled with a precipitated white product which, in some cases, made difficult to identify the crack position.

In Table 3, the comparison between the crack width before and after the treatments is included.

Results concerning the reduction of crack width corroborate those observed with the indirect measure of electrical resistance (see Figure 2), confirming the higher efficiency of electromigration and capillary suction treatments compared to the injection method. Moreover, the importance of the crack size on the treatment effectiveness can be also deduced from Table 3, with higher sealing ability obtained for the smallest crack sizes. However, it should be noticed that too narrow cracks, such as M1, can be counterproductive for the treatment effectiveness, probably due to higher difficulties for the product transport.

### 3.3. Interaction between Colloidal Nanosilica and Treated Mortar

Different samples with different effectiveness of crack sealing were subjected to a three-point flexural bending test to characterize their residual mechanical behaviour after the 15-days conditioning period under humid conditions. The evaluated samples were high effectiveness (S1), average effectiveness (M1, S2), and low effectiveness (I1). The obtained results showed a quite low residual mechanical behaviour as most of the samples broke with a smaller load than the minimum value detected by the test machine. Sample M1, the one with the smallest crack width subjected to electromigration, showed a certain load (0.35 kN) but considerably smaller than the initial one (2.1 kN), and the resulting crack was formed on the previous treated one. This could indicate that longer conditioning times (>15 days) are probably needed.

The type of interaction established between the silica nanoparticles and the mortar matrix of the crack walls has been analyzed by BSEM/EDX. Two different samples, M2 and I2, have been studied to assess the influence of the transport mechanism as electromigration (M2 sample) was proved to be the most effective treatment and injection (I2) the least effective one. Moreover, possible relations between the sealing effectiveness and the interaction between the nanoparticle and the mortar matrix were evaluated.

**Figure 4:**
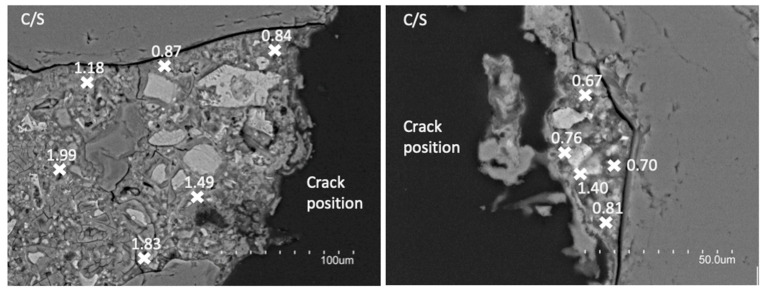
BSEM images of M2 sample after the electromigration treatment. C/S ratios of the C-S-H gels are indicated.

**Figure 5:**
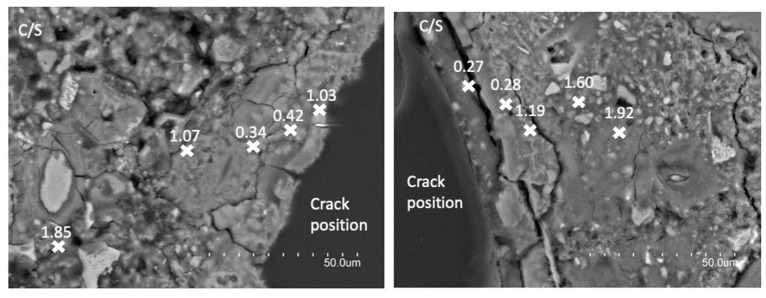
BSEM images of I2 sample after the injection treatment. C/S ratios of the C-S-H gels are indicated.

Figure 4 shows two BSEM images of M2 sample and Figure 5 two BSEM images of I2 sample, taken after the 15-days conditioning treatment in each case. The images show cross-section of the repaired cracks but cutting the sample just following the crack. The modifications occurred in the C/S ratios of the C-S-H gels formed from the crack to 50–100 μm inwards, which are also indicated. An image of each side of the crack is shown for each sample.

The obtained results seem to demonstrate that the evaluated treatments influence the cement paste microstructure in a different way. Therefore, this different influence could explain the different effectiveness of each treatment. In the case of the electromigration treatment (sample M2; see Figure 4), the most effective one, the C/S ratios measured in the C-S-H gels near the treated crack vary between 0.7 and 1.5. However, the C/S ratio of C-S-H in conventional Portland cement pastes varies from 1.2 to 2.3 [26], with a mean value around 1.75. This “conventional” C/S ratio is obtained in the M2 sample only in zones more than 50 μm far from the crack, whereas in those near the crack the measured C/S ratio is quite lower. This must be related with a pozzolanic reaction promoted by the colloidal silica nanoparticles used, with the subsequent consumption of portlandite. These reaction processes should be similar than those described for OPC cementitious materials with the inclusion of high mineral admixtures contents with high silica percentage in their composition [27,28,29]. In the case of nanosilica particles, the reaction of silica nanoparticles with the existing C-S-H gels producing C-S-H gels with reduced C/S ratio has already been reported [23]. The C-S-H gels with lower C/S are typically considered to be formed by longer length chains of tetrahedral silica [30]. Thus, the chemical reaction between the nanosilica and the portlandite of the cementitious matrix could be expected, forming new C-S-H gels enriched in silica. Moreover, even chemical reaction of the previous C-SH gels could also occur but this aspect must be evaluated in depth. In this sense, similar type of chemical reaction has been detected when using expansive cementitious grouts for crack sealing [10].

On the contrary, this phenomenon is not so evident in the I2 sample. In this case, the C/S ratios obtained near the crack are very low, varying between 0.25 and 0.45. These values are too low to be measured in C-S-H gels. Therefore, in the measured zones, the high silica content measured must be due to the presence of unreacted nanosilica particles. Only in deeper areas from the crack mouth some C/S ratios such as those measured in M2 sample but a little bit higher (between 1.0–1.2) are detected. Thus, the existence of reactions similar to those described in the previous case but with lower effectiveness or importance can be expected.

Considering the obtained results and taking into account the higher proved effectiveness of the electromigration treatment with respect to the injection one, it seems that the effectiveness of the treatment is at least partially related to the existence of reactions between the colloidal nanosilica particles and the cement paste matrix. Therefore, not only a physical action of the colloidal nanosilica particles but also a chemical one occurs, and thus, not only the sealing of the crack but a certain healing process can be expected.

## 4. Conclusions

Colloidal nanosilica has been successfully applied as a repair method for sealing cracks in mortar samples. The influence of the nanoparticle transport mechanism in the effectiveness of the treatment has been assessed: migration, injection, and capillary suction mechanisms has been considered. A better performance with the electromigration of the nanoparticles has been found. The crack size has also shown to be a critical parameter, with better effectiveness of the treatment for smaller cracks. In the case of sufficiently small cracks, a sealing efficiency of 100% has been achieved.

A chemical reaction between the nanosilica particles and the existing treated cement matrix can be deduced from BSEM/EDX results. C-S-H gels with lower C/S ratio are formed on the crack mouth. The higher effectiveness of electromigration treatment could be related to a higher development of this chemical interaction between the silica nanoparticles and the existing cement matrix. The C/S ratio of the new C-S-H gels formed, between 0.7–1.5, could be due to the pozzolanic reaction of the silica gel with the portlandite. Moreover, the interaction of the previous C-S-H gels with the silica nanoparticles forming new C-S-H gels with lower C/S ratio cannot be ruled out. Other less effective methods, such as injection, showed the presence of silica gel in the crack, but very low C/S are measured, indicating a less developed reaction/interaction between the nanosilica and the calcium of the matrix.

## Figures and Tables

**Table 1:** Chemical composition of CEM I 42.5 R/SR (% oxides in weight) (XRF).

CaO	SiO_2_	Al_2_O_3_	Fe_2_O_3_	MgO	K_2_O	Na_2_O	SO_3_
60.3	17.4	4.68	5.08	1.78	0.34	0.18	3.17

**Table 2:** Mortar samples, crack width after the three-point bending test, and method for colloidal nanosilica application.

Sample	Crack Width/μm	Method
M1	49 ± 7	Electromigration (M)
M2	166 ± 20	Electromigration (M)
M3	248 ± 40	Electromigration (M)
I1	237 ± 12	Injection (I)
I2	395 ± 13	Injection (I)
S1	60 ± 8	Capillary suction (S)
S2	94 ± 24	Capillary suction (S)
S3	180 ± 36	Capillary suction (S)

**Table 3:** Crack width measured before and after the sealing treatments and reduction percentage obtained in each case.

Sample	Initial Crack Width/μm	Final Crack Width/μm	% Reduction
M1	49 ± 7	36 ± 6	25
M2	166 ± 20	86 ± 32	48
M3	248 ± 40	246 ± 23	1
I1	237 ± 12	220 ± 39	7
I2	395 ± 13	299 ± 31	24
S1	60 ± 8	NEGLIGIBLE	100
S2	94 ± 24	47 ± 24	50
S3	180 ± 36	143 ± 11	21
